# 18. An Easy-to-Implement Clinical-Trial Frailty Index Based on Accumulation of Deficits: Validation in Zoster Clinical Trials

**DOI:** 10.1093/ofid/ofab466.220

**Published:** 2021-12-04

**Authors:** Melissa K Andrew, Sean Matthews, Joon Hyung Kim, Megan Riley, Desmond Curran

**Affiliations:** 1 Dalhousie University, Halifax, Nova Scotia, Canada; 2 Freelance c/o GSK, Wavre, Brabant Wallon, Belgium; 3 GSK, Rockville, Maryland

## Abstract

**Background:**

The impact of frailty on the efficacy and safety of vaccines and therapeutic interventions is increasingly recognized, yet assessment of frailty in clinical trials is often considered logistically challenging. We developed the retrospective Clinical Trial Frailty Index (CT-FI), using baseline medical history and patient reported outcomes collected via standard instruments (Short Form Survey-36 and Euro Quality of Life-5 Dimension) in two clinical trials of the adjuvanted recombinant zoster vaccine (RZV, ZOE-50 [NCT01165177] and ZOE-70 [NCT01165229]). This post-hoc analysis aimed to show that CT-FI is a robust measure that may be used in any analysis where sufficient patient data has been collected in a clinical trial.

**Methods:**

Items included in the CT-FI were scored from 0 to 1, summed for each participant and divided by the total number of potential deficits. CT-FI was validated using descriptive methods verifying distribution and age- and sex-associations in relation to established FI characteristics, Cox regressions in relation to fatal outcomes hypothetically related to frailty, and re-sampling methods (Jackknife and Bootstrap procedures) within the FI to demonstrate robustness to inclusion/exclusion of specific individual variables.

**Results:**

The CT-FI distribution followed a gamma distribution with a range of 0 to 0.695; the distribution shifted to the right with age. The age-related slope of mean deficit accumulation per year increased with chronological age and was higher for women than men. The rate of mean deficit accumulation was 0.0025 for women vs 0.0016 for men < 70 years of age, and this increased to 0.0058 for women vs 0.0047 for men ≥70 years of age. In univariate and multivariate Cox regression survival analyses, FI, chronological age and sex were significant predictor factors for mortality. The Jackknife and Bootstrap re-sampling methods showed that the performance of CT-FI was not sensitive to inclusion/exclusion of specific individual or groups of variables, demonstrating the robustness of this methodology.

**Conclusion:**

The current analysis validates that CT-FI, an easy-to-implement FI, is a robust method which allows retrospective/prospective evaluation of clinical outcomes by frailty status in clinical trials.

**Disclosures:**

**Melissa K. Andrew, MD, PhD**, **GSK** (Grant/Research Support)**Pfizer** (Grant/Research Support, Advisor or Review Panel member)**Sanofi** (Consultant, Grant/Research Support, Advisor or Review Panel member)**Seqirus** (Advisor or Review Panel member) **Sean Matthews, MSc**, **GSK** (Independent Contractor) **Joon Hyung Kim, MD**, **GSK group of companies** (Employee, Shareholder) **Megan Riley, PhD**, **GSK group of companies** (Employee, Shareholder) **Desmond Curran, PhD**, **The GSK group of companies** (Employee, Shareholder)

